# Wernicke’s Encephalopathy as a Rare Complication of Hyperemesis Gravidarum: A Case Report and Review of Literature

**DOI:** 10.7759/cureus.2597

**Published:** 2018-05-09

**Authors:** Elanagan Nagarajan, Chintan Rupareliya, Pradeep C Bollu

**Affiliations:** 1 Department of Neurology, University of Missouri, Columbia, USA; 2 Department of Neurology, University Of Kentucky College of Medicine

**Keywords:** nystagmus, hyperemesis gravidarum, wernicke's encephalopathy, encephalopathy, ataxia, pregnancy complications, rare complications of pregnancy, vomiting during pregnancy

## Abstract

Wernicke's encephalopathy (WE) is a rare neurological syndrome that presents in the setting of thiamine deficiency. Though alcoholism is the most common cause of this condition, a few other etiologies include malnutrition from other causes, hemodialysis, and hyperemesis gravidarum. In this case report, we aim to report a case of a young woman who developed WE in the setting of hyperemesis gravidarum (HG) that improved with thiamine replacement. This manuscript details her presentation and clinical examination and includes a spontaneous upbeat nystagmus and goes over the condition along with a review of the literature.

## Introduction

Wernicke's encephalopathy (WE) is a devastating, acute neurological condition due to thiamine deficiency [1]. The classic triad of WE includes confusion, oculomotor dysfunction, and ataxic gait. The presence of one or two core manifestations in the appropriate clinical setting confirms the diagnosis of WE [1]. The incidence of WE ranges anywhere between 0.2% and 2.8% of the general population in western countries [1]. Among them, a majority of the affected patients have a known history of chronic alcoholism. Other potential causes include malnutrition, malignancy, gastric bypass surgery, hemodialysis, and hyperemesis gravidarum; they are very rare in the western world [1-2]. We are presenting a case of pregnant women with HG that developed the classical clinical triad of WE along with spontaneous nystagmus and typical imaging findings on the brain imaging.

## Case presentation

A 36-year-old Caucasian female, 16 weeks into her pregnancy, presented with intractable nausea, non-bloody emesis, and poor oral intake for the prior two months. She initially presented to an outside facility for an evaluation of loss of consciousness (LOC) and mild abdominal pain. The LOC was found to be secondary to syncopal episode. The initial blood workup was significant for leukocytosis (11.54L), hyponatremia (130 mmol /L), and hypokalemia (2.5 mmol /L). Her aspartate aminotransferase (AST) was 496 U/L, alanine aminotransferase (ALT) was 1280 U/L, and alkaline phosphatase (ALP) was 76 U/L. Lipase levels were also elevated to 83 U/L. Urine analysis was significant for ketonuria. The patient was transferred to our tertiary care facility for the management of electrolyte imbalance and acute pancreatitis with a presumed diagnosis of HG. She had an ultrasound (US) of the abdomen, which revealed mild hepatic steatosis. The patient was treated symptomatically with antiemetics, pain medications, and intravenous (IV) fluids. She had poor oral intake during the hospital stay, and her blood sugar was in the range of 50-70 mg/dL. She was started with intravenous (IV) dextrose to treat her hypoglycemia. Within 48 hours after starting IV dextrose, she developed confusion and started having problems with learned memories. She also complained of blurry vision and horizontal double vision that was worse when she turned her head toward the right side. She also had a subjective sensation of constant somersaulting and reported well-formed visual hallucinations. Her physical examination showed spontaneous upbeat nystagmus (Video 1), gait instability, and ataxia with a tendency to lean to the left side while walking.

**Video 1:** Initial physical examination revealed spontaneous upbeating nystagmus

**Figure 1:**
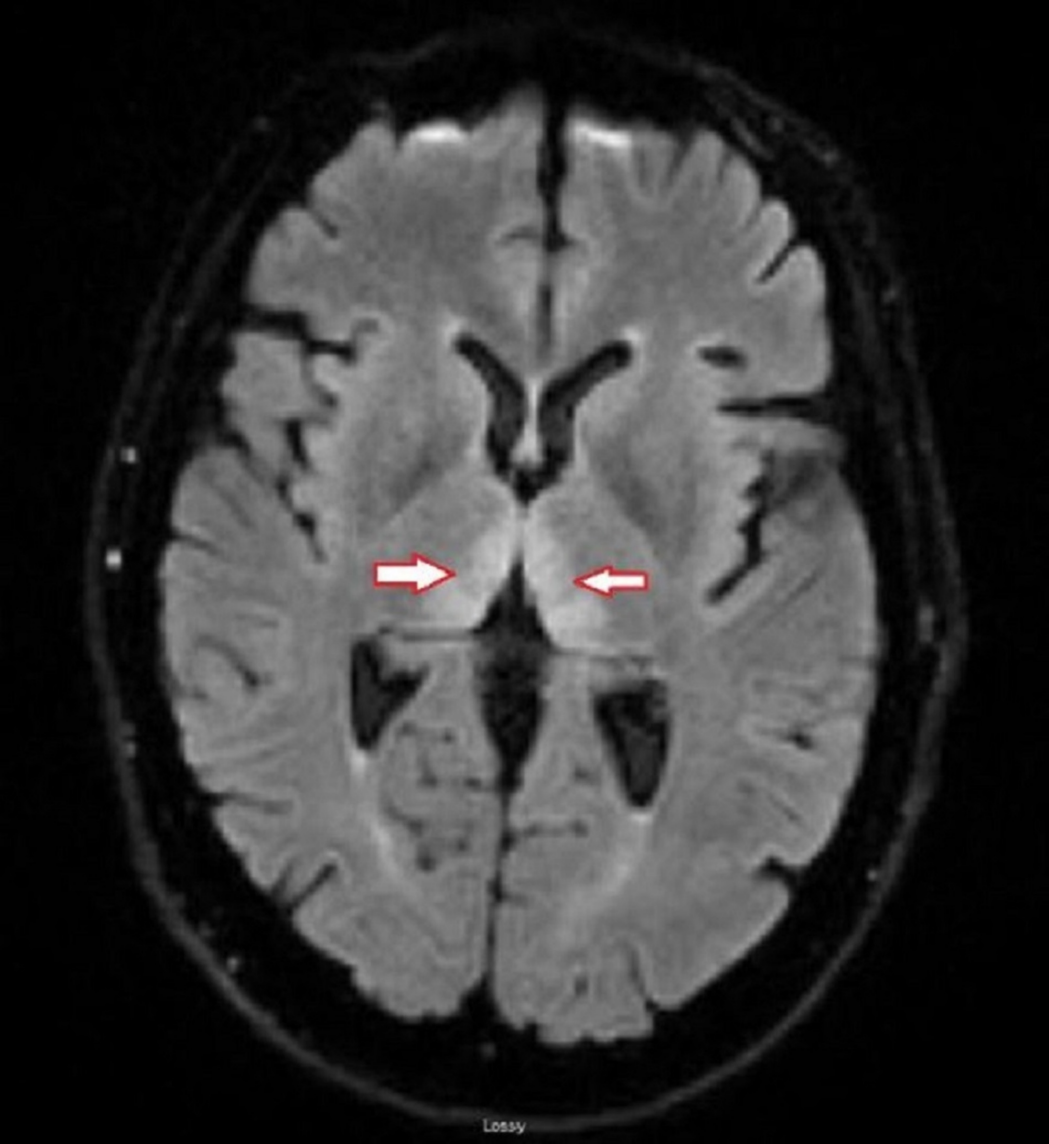
T2/FLAIR sequence of the axial view of the MRI brain shows the bilateral thalamic hyperintensities (red and white arrows) Abbreviations: FLAIR = fluid attenuation inversion recovery; MRI = magnetic resonance imaging

**Figure 2:**
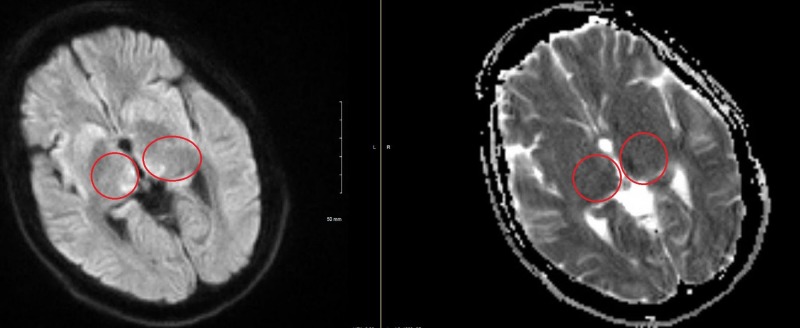
DWI MRI of brain shows cytotoxic edema on bilateral thalamic regions with corresponding ADC changes significantly on the left when compared to right (red circles) Abbreviations: DWI = diffusion restriction imaging; MRI = magnetic resonance imaging; ADC = apparent diffusion coefficient

Magnetic resonance imaging (MRI) of the brain, as shown in Figure 1, revealed symmetrical T2/fluid attenuated inversion recovery (FLAIR) hyperintense signals on bilateral medial thalamic regions, more pronounced on the left along with a diffusion restriction in the same regions. Figure 2 shows an axial view of the diffusion-weighted imaging (DWI) sequence, which revealed cytotoxic edema in regions of the bilateral thalamus with corresponding apparent diffusion coefficient (ADC) changes.

She also underwent a magnetic resonance venogram (MRV) that did not show venous sinus thrombosis. Blood was drawn for thiamine levels before the replacement, which showed a low thiamine level of 40 nmol/L (normal range: 70-180 nmol/L). She was treated with high dose IV thiamine 400 mg for three days followed by 100 mg per day. At the time of discharge, her memory and gait instability improved significantly. However, the patient had persistent upbeating nystagmus. She was discharged to inpatient rehabilitation with daily oral thiamine replacement. The patient was re-evaluated from three months of diagnosis in our outpatient clinic and was noted to have a persistent upbeating nystagmus (Video 2). She no longer had any major deficits in memory and/or gait.

**Video 2:** An eye exam at three months' follow-up visit The patient is noted to have persistent upbeating nystagmus.

**Figure 3:**
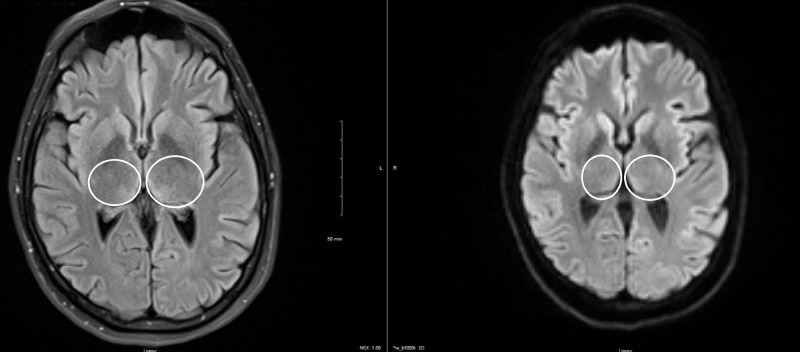
Follow-up images at three months Axial view of T2/FLAIR sequences shows the interval resolution of the previously seen hyperintense signal in bilateral thalamic regions (white circles) Abbreviations: FLAIR = fluid attenuation inversion recovery

We also obtained a repeat MRI of the brain during this follow-up that showed a significant resolution of previously identified bilateral hyperintense signals in bilateral medial thalamic regions on T2/FLAIR sequence (Figure 3). As shown in Video 3, at six months follow-up, her spontaneous vertical nystagmus had resolved, but she continued to have gaze-evoked horizontal and vertical nystagmus.

**Video 3:** An eye exam at the six months' follow-up visit Spontaneous vertical nystagmus has resolved, but she continued to have gaze-evoked horizontal and vertical nystagmus.

## Discussion

HG is one of the most common causes of hospitalization during the first half of pregnancy. In the United States, it’s prevalence ranges anywhere between 0.3-3% of pregnant woman and it is more common in non-Caucasian and nonsmokers when compared to Caucasians and smokers [3]. The mechanism of the disease and causes still remain controversial [3]. HG is a diagnosis of exclusion and all other potential causes need to be excluded. Along with severe repeated vomitings, these patients have electrolyte abnormalities, elevated liver enzymes, dehydration, orthostatic hypotension and sometimes, mental status changes [4]. The repeated vomitings and poor nutrition during HG causes both water and fat-soluble vitamin deficiency including vitamin B1 (also known as thiamine) and K deficiency [4-5].

Thiamine is a water-soluble vitamin and is absorbed throughout the small intestine and more efficiently in the upper jejunum [6]. Thiamine acts as a cofactor for enzymes such as transketolase, alpha-ketoglutarate dehydrogenase, and pyruvate dehydrogenase which play a major role in carbohydrate metabolism [7]. Thiamine also has a protective effect on retinal neurons against glutamate toxicity and aids in the survival of hippocampal neurons in vivo studies. It also facilitates neurotransmission and the release of neurotransmitters such as acetylcholine, dopamine, and norepinephrine [8]. During HG, thiamine storage is depleted and the absorption rate is decreased significantly due to excessive vomiting, poor oral intake, and high metabolic demand. In our patient, we believe that the rapid correction of hypoglycemia without any replacement of thiamine caused the depletion of residual storage.

The classic triad of WE is often seen in patients with HG as compared to those with WE associated with alcoholism [7]. Confusion is the most common findings followed by nystagmus and gait instability. In our patient, the etiology of the gait instability is multifactorial and due to a combination of ocular dysfunction, poor nutritional status, orthostatic intolerance, and other dysfunctions of other, higher structures. Our patient had a classic clinical picture in the appropriate setting, and the diagnosis was confirmed by the low levels of Thiamine. Patients with WE show a selective pattern of damage in the subcortical regions of the brain that can involve the thalamus, mammillary bodies, midbrain, and brainstem structures, including the vestibular system. The exact pathophysiology of the selective pattern of neurodegeneration remains unclear [9]. Reversible cytotoxic edema is noted in the subcortical region and predominately seen in the T2/FLAIR and diffusion-weighted imaging (DWI) sequences [10]. Our patient had bilateral thalamic hyperintensities on FLAIR and DWI changes significantly on the left side as compared to the right. The repeat MRI scan during her follow-up showed the resolution of previously seen imaging findings. Thiamine replacement should always precede the replacement of sugars during the treatment of WE. A low threshold of suspicion and quick intervention are keys to the prevention of significant neurological injury. The dosage and duration of thiamine replacement are not well defined in these patients.

## Conclusions

WE is one of the rare but potentially reversible neurological complications during pregnancy due to hyperemesis gravidarum. In our patient, the replacement of sugar without thiamine is the likely precipitant of WE. Therefore, in pregnant women with frequent vomiting and any neurological changes, WE should be considered and promptly treated. The replacement of electrolytes along with thiamine warranted the prevention of permanent neurological deficits.

## References

[1] Harper C, Fornes P, Duyckaerts C, Lecomte D, Hauw JJ (1995). An international perspective on the prevalence of the Wernicke-Korsakoff syndrome. Metab Brain Dis.

[2] Galvin R, Bråthen G, Ivashynka A, Hillbom M, Tanasescu R, Leone MA (2010). EFNS guidelines for diagnosis, therapy and prevention of Wernicke encephalopathy. Eur J Neurol.

[3] London V, Grube S, Sherer DM, Abulafia O (2017). Hyperemesis gravidarum: a review of recent literature. Pharmacology.

[4] Lee NM, Saha S (2011). Nausea and vomiting of pregnancy. Gastroenterol Clin North Am.

[5] Baba Y, Morisawa H, Saito K, Takahashi H, Rifu K, Matsubara S (2016). Intraperitoneal hemorrhage in a pregnant woman with hyperemesis gravidarum: vitamin K deficiency as a possible cause. Case Rep Obstet Gynecol.

[6] Frank LL (2015). Thiamin in clinical practice. JPEN J Parenter Enteral Nutr.

[7] Ashraf VV, Prijesh J, Praveenkumar R, Saifudheen K (2016). Wernicke's encephalopathy due to hyperemesis gravidarum: clinical and magnetic resonance imaging characteristics. J Postgrad Med.

[8] Berg JM, Tymoczko JL, Stryer L (2002). Biochemistry: 5th Edition. https://www.ncbi.nlm.nih.gov/books/NBK21154/.

[9] Hazell AS, Butterworth RF (2009). Update of cell damage mechanisms in thiamine deficiency: focus on oxidative stress, excitotoxicity and inflammation. Alcohol Alcohol.

[10] Suzuki S, Ichijo M, Fujii H, Matsuoka Y, Ogawa Y (1996). Acute Wernicke's encephalopathy: comparison of magnetic resonance images and autopsy findings. Intern Med.

